# Early Stage Combination Treatment with Methylprednisolone Pulse and Remdesivir for Severe COVID-19 Pneumonia

**DOI:** 10.3390/ijerph20021081

**Published:** 2023-01-07

**Authors:** Claudio Mastruzzo, Elena Commodari, Umberto Grasso, Valentina Lucia La Rosa, Daniela Balsamo, Cristina Circo, Rosario Oliveri

**Affiliations:** 1 Unit of Respiratory Diseases, Department of Medicine, Garibaldi Hospital, 95124 Catania, Italy; 2 Department of Educational Sciences, University of Catania, 95124 Catania, Italy; 3 Unit of Internal Medicine, Department of Medicine, Garibaldi Hospital, 95124 Catania, Italy

**Keywords:** COVID-19, remdesivir, corticosteroids, pulse therapy

## Abstract

Background: This study evaluated the clinical outcomes of patients with severe COVID-19 pneumonia treated with remdesivir plus standard corticosteroid treatment (SCT) or with remdesivir plus high-dose corticosteroid pulse therapy (HDCPT). Methods: One hundred and two patients with severe COVID-19 pneumonia and respiratory failure were included. The patients were divided into two cohorts. The first comprised patients who received remdesivir and SCT, consisting of 6 mg dexamethasone daily for up to 10 days or until hospital discharge. The second included patients who received remdesivir and HDCPT, composed of 250 mg iv of methylprednisolone for three days, followed by a slow reduction in the dose of steroids. The severity of hypoxemia was assessed using the SaO2/FiO2 peripheral oxygen saturation index. Results: 55 received remdesivir plus HDCPT, and 47 received remdesivir plus SCT. Mortality at 30 days was significantly lower among patients who received remdesivir plus HDCPT (4/55) than among those who did not (15/47). In patients who received remdesivir plus HDCPT, 7.3% required invasive mechanical ventilation and admission to the ICU and 36.4% non-invasive ventilation versus 29.8% and 61.7%, respectively, among those treated with remdesivir plus SCT. Remdesivir plus HDCPT induced a significantly faster improvement in the SaO2/FiO2 index. Conclusion: Early combination treatment with remdesivir plus HDCPT reduced in-hospital mortality and the need for admission to the ICU. Furthermore, it improved the SaO2/FiO2 index faster in patients with severe COVID-19 pneumonia.

## 1. Introduction

The clinical manifestation of the COVID-19 disease may vary substantially. Most infected people develop mild to moderate illness and recover without hospitalization; however, some patients present severe COVID-19 pneumonia that requires oxygen therapy or mechanical ventilation with significant morbidity and mortality. It is believed that uncontrolled virus reproduction leads to a hyper-inflammation phase with a pro-inflammatory cytokine storm and high inflammatory products resulting in immunopathological lung injury, diffuse alveolar damage with the development of acute respiratory distress syndrome (ARDS), and death [1]. Severe pneumonia and acute respiratory distress syndrome are the leading causes of death in patients with COVID-19 [2].

Although specific guidelines are available for COVID-19 patients [3,4,5], actual treatment may not be sufficient to control disease progression and avoid worse outcomes. Several clinical trials evaluated the efficacy of remdesivir as antiviral therapy, showing its efficacy on clinical outcomes of hospitalized patients with COVID-19 [6,7]. However, antiviral treatment alone may not control disease development and avoid inferior outcomes, as considerable morbidity and mortality due to COVID-19 persist.

Emerging evidence suggests that the severity of the disease may be due to a dysregulated inflammatory response associated with elevated cytokine levels. Therefore, immunosuppressive therapy may protect against COVID-19 infection by preventing or reducing excessive immune reactions that may drive clinical deterioration. Corticosteroids are powerful anti-inflammatory drugs that inhibit multiple pathways in the inflammatory process and can play a critical role in reducing dysregulated inflammation in the respiratory system, preventing the induction of cytokine storms and ARDS. Favorable data have emerged to support the use of corticosteroids in patients with severe COVID-19 [8,9,10], and glucocorticoids are indicated in the current guidelines [3,4,5]. However, the optimal dose and timing of steroids are not yet precise, and the impact of corticosteroid therapy on the outcomes of patients with COVID-19 is highly controversial.

Several pieces of evidence suggest that early steroid pulse may have relevant survival benefits [11,12,13,14] in patients with severe COVID-19, preventing the progression of the disease to a critical stage and improving clinical outcomes.

Therefore, we hypothesized that in patients with severe COVID-19 pneumonia, early combination treatment with pulses of remdesivir and methylprednisolone that aim to inhibit viral replication and attenuate excessive systemic inflammatory responses might obtain relevant clinical benefits, accelerate disease resolution and reduce the need for invasive mechanical ventilation, admission to the ICU, and in-hospital mortality. To address this problem, we performed a retrospective cohort study evaluating the clinical results of patients with severe COVID-19 pneumonia treated with remdesivir plus high-dose steroid treatment compared with patients treated with remdesivir plus standard steroid treatment.

## 2. Materials and Methods

### 2.1. Study Design and Patients

This single-center retrospective cohort study was carried out at Garibaldi Hospital, Catania, Italy. From 10 October 2020 to 31 May 2021, 359 consecutive patients with a clinical diagnosis of COVID-19 were admitted to our hospital, and 102 patients with confirmed COVID-19 pneumonia matched our study’s inclusion and exclusion criteria. Patients who died within two days after hospital admission were excluded to avoid the influence of early mortality before presenting the efficacy of treatment. Up to 30 days after hospital admission, all patients were tracked until death, ICU admission, or discharge. Clinical signs and radiographs were regularly evaluated. Laboratory tests were recorded, including complete blood count (CBC), C-reactive protein (CRP), and other inflammatory markers. The severity of hypoxemia was assessed using the SaO2/FiO2 peripheral oxygen saturation index. SaO2/FiO2 has shown a robust linear relationship in moderate to severe ARDS. Previous large studies support the use of SaO2/FiO2 as a practical alternative to PaO2/FiO2 and a useful diagnostic tool for clinical practice and enrollment in clinical trials [15,16]. The patients were classified into mild, moderate, and severe by applying Rice et al. cut-offs [17]. The need for oxygen therapy (nasal cannula, oxygen mask, and reserve mask) and non-invasive ventilation (NIV) was recorded. The modified age Charlson Comorbidity Index was used to assess the occurrence of comorbidities.

### 2.2. Inclusion Criteria

Patients were eligible if they matched the following criteria: (1) aged 18 years of age or older; (2) COVID-19 pneumonia confirmed by a viral pneumonia radiographic pattern and by positive SARS-CoV-2 PCR; (3) and respiratory failure and need for oxygen therapy, with a SaO2/FiO2 index of <315.

### 2.3. Exclusion Criteria

Patients were not eligible for enrollment if they satisfied the following criteria: (1) onset of symptoms within ten days; (2) requirement for non-invasive ventilation (NIV); (3) positive procalcitonin; (4) hepatitis B and C viruses; (5) and uncontrolled hypertension, uncontrolled diabetic mellitus, history of gastrointestinal bleeding, acute heart failure, history of active malignancies, or prolonged immunosuppressive therapy.

### 2.4. Procedures

All patients received remdesivir with a five-day regimen (200 mg the first day, 100 mg for the other four days). The institutional hospital COVID-19 procedures for corticosteroids were designed by consensus based on current national and international recommendations [3,5] and the best available data in the literature at the time of the study [9,11]. They recommended the use of corticosteroids in patients with COVID-19 pneumonia and respiratory failure in two possible treatment regimens at the discretion of the treating medical team for each patient: (a) standard corticosteroid therapy (SCT) consisting of dexamethasone 6 mg daily for up to 10 days or until hospital discharge, whichever comes first; (b) or high-dose corticosteroid pulse therapy (HDCPT) consisting of methylprednisolone (MP) 250 mg iv for the first three days followed by MP 40 mg on days 4–8 and then slowly decrease every three days of the dose of steroids. The attending physician chose the patient’s treatment plan. All patients received antithrombotic treatment with sodium enoxaparin, antibiotic agents to prevent bacterial infection, vitamin D supplements, and treatment for preexisting comorbidities. Non-invasive mechanical ventilation was indicated in case of dyspnea or whenever SaO2 was <90% despite oxygen therapy. ICU admission and invasive mechanical ventilation were indicated if, despite non-invasive ventilation, any of the following were present: PaO2/FiO2 < 100 mmHg, dyspnea, and a respiratory rate greater than 40 breaths per minute. All patients were monitored for adverse effects (e.g., hyperglycemia, secondary infections, psychiatric effects, and reactivation of latent infections), and drug–drug interactions were evaluated. The Ethics Committee of the Garibaldi Hospital approved the study (n. 443/C.E., 27 June 2022), and participants filled out and signed the informed consent after recovering from severe COVID-19 pneumonia.

### 2.5. Outcomes

The primary endpoint in both groups was mortality at 30 days. Other outcome measures were: (a) clinical improvement as the SaO2/FiO2 index on day 5 (SaO2/FiO2_5_) and 10 (SaO2/FiO2_10_); (b) admission to the intensive care unit (ICU), and the necessity for invasive mechanical ventilation (IMV); (c) or the necessity for non-invasive mechanical ventilation (NIV).

### 2.6. Statistical Analysis

Statistical analyses were conducted using the Statistical Package SPSS version 27.0 (IBM Corp., Armonk, NY, USA). Means, standard deviation, and *t*-test analyses were calculated to assess homogeneity for age, sex, number of comorbidities, and basal SaO2/FiO2 (SaO2/FiO2_0_) between the group of patients with remdesivir plus SCT and the group of patients with remdesivir plus HDCPT and the presence of significant differences in SaO2/FiO2_5_ and SaO2/FiO2_10_ after treatment. The Chi-square test measured differences in frequencies and percentages for death, admission to the intensive care unit with the need for IMV, and the need for NIV in the two groups of patients. To better analyze the impact of the two types of treatment on primary outcomes (death, admission to the ICU, need for NIV, SaO2/FiO2_5_, SaO2/FiO2_10_), with a control for age and the number of comorbidities, several multiple regression analyses were performed. 

## 3. Results

### 3.1. Patients

The study involved 102 patients, 61 (59.8%) of whom were men, with the remaining being women. The median age of the sample was 64.05 years (SD 13.3 years). Fifty-five patients (53.9%) received remdesivir plus HDCPT, and 47 (46.1%) received remdesivir plus standard corticosteroid therapy (SCT). *T*-test analysis verified that the two groups of patients (remdesivir plus HDCPT group and remdesivir plus SCT group) were homogeneous for age and sex, the number of comorbidities, and SaO2/FiO2_0_ (Table 1). 

### 3.2. Mortality

**Figure 1:**
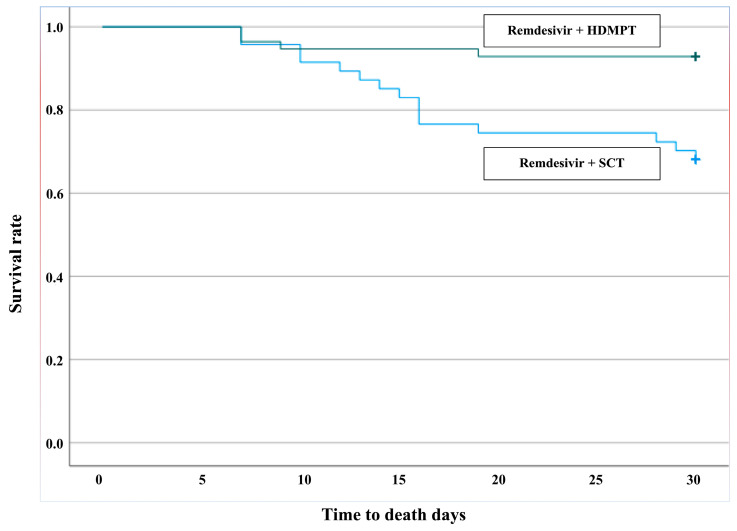
Kaplan–Meier estimator of the survival rate between the remdesivir plus high-dose corticosteroid pulse therapy (HDCPT) and remdesivir plus standard corticosteroid therapy (SCT) groups. Log-rank: 9.99, *p* = 0.002.

A total of 31.9% (15/47) of the patients who received remdesivir plus SCT died 30 days after admission compared with 7.3% (4/55) of the patients who received remdesivir plus HDCPT. Chi-square values (X^2^ = 10.15, sig: *p* < 0.001) and the Kaplan–Meier curve (log-rank: 9.99, *p* = 0.002) showed a significant difference in mortality at 30 days in the two groups of patients, with higher survival in patients treated with remdesivir plus HDCPT (Figure 1).

### 3.3. Admission to the ICU and IMV

Chi-square analysis showed a significant association between treatment typology and admission to the ICU with IMV (X^2^ = 8.84, *p* < 0.05). Only 7.3% (4/55) of the patients who received remdesivir plus HDCPT required access to the ICU and IMV compared with 29.8% (14/47) of the patients who received remdesivir plus SCT (Table 2). Regarding NIV, the Chi-square values (X^2^ = 11.05, *p* < 0.001) showed a significant difference in the need for NIV in the two groups of patients. A total of 30.9% (17/55) of patients who received remdesivir plus HDCPT required NIV compared with 63.8% (30/47) of patients who received remdesivir plus SCT (Table 2).

### 3.4. Values of SaO2/FiO25 and SaO2/FiO210 in the Two Groups of Patients

**Figure 2:**
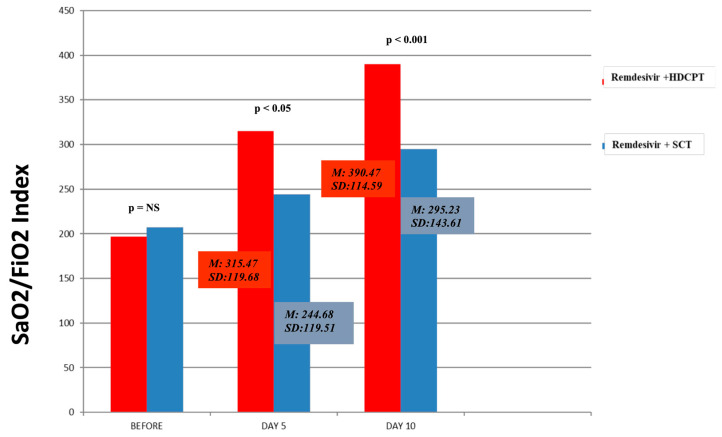
Mean SaO2/FiO2 index before and after 5 and 10 days of therapy in the remdesivir + HDMPT and remdesivir + SCT groups. Abbreviations: SCT: standard corticosteroid therapy; HDCPT: high-dose corticosteroid pulse therapy.

The differential descriptive analyses for treatment revealed significant variations in the values of SaO2/FiO25 and SaO2/FiO210 after treatment between the two patient groups. In particular, remdesivir plus HDCPT had higher SaO2/FiO2 values on days 5 and 10 compared with the remdesivir plus SCT group, as shown in Figure 2 (SaO2/FiO2_5_: remdesivir plus HDCPT: M = 315.47, SD = 119.68; remdesivir plus SCT: M = 244.68, SD = 105.51, t = −3.13, *p* < 0.05; SaO2/FiO2_10_: remdesivir plus HDCPT: M = 390.47, SD = 114.59, remdesivir plus SCT: M = 295.23, SD = 143.61, t = −3.72 *p* < 0.001).

### 3.5. Regression Analyses

To better evaluate the impact of typology treatment on 30-day mortality, admission to the ICU, NIV, and SaO2 on days five and ten, we performed multiple regression analyses using the “entry forced” method. The preliminary correlation matrix showed the absence of correlation values that can hinder the correct interpretation of the regression results. Moreover, all correlation coefficient values were less than 0.70. The value of 0.70 is the one that causes problems in the interpretation of regression analyses. Furthermore, there was no evidence of multicollinearity between predictors in any regression analyses (VIF < 2; r’s < 0.60). The type of treatment, age, and the number of comorbidities were the independent variables; SaO2/FiO25, SaO2/FiO210, admission to the ICU, NIV, and death were the dependent variable. Regression analyses allowed the evaluation of the impact of treatments, age, and the number of comorbidities on primary outcomes. Although causal relationships between the main variables are challenging to establish, the results of the regression analyses showed that the treatment typology significantly affected all the dependent variables (30-day mortality, admission to the ICU, NIV, and SaO2/FiO2 at five and ten days). Another significant variable, but less important than treatment, in determining only SaO2/FiO2 at five and ten days was age. However, this variable did not affect 30-day mortality, admission to ICU, and NIV. This result confirms the significant role of treatment typology on all outcomes. The number of comorbidities did not contribute to the values of the dependent variables (Table 3).

In particular, regression analyses showed the following results:

(a) *Thirty-day mortality*: The treatment typology was the significant predictor of mortality. This variable explained much of the variation in the dependent variable (F = 7.55, *p* < 0.001; R^2^ = 0.18; treatment: β = −0.31). The treatment typology significantly affected mortality in the sample;
(b) *ICU admission and NIV*: The treatment typology explained much of the variation in the ICU admission variable (F = 4.25, *p* < 0.05; R^2^ = 0.11; treatment: β = −0.29, t = −3.06, *p* < 0.05). Treatment was also a significant predictor of the need for NIV (F = 5.66, *p* < 0.001; R^2^ = 0.48; β = −0.32);
(c) *SaO2/FiO2_5_*: Treatment and age were significant predictors of SaO/FiO2_5_ (F = 5.51, *p* = 0.002; R^2^ = 0.14). The t-statistic and β values that allow us to determine the relative importance of each variable in the model showed that the treatment typology and age explained a large part of the variation in the dependent variable (treatment: β = 0.28, t = 3.04, *p* = 0.003; age: β = −0.34, t = −2.46, *p* = 0.01);
(d) *SaO2/FiO2_10_*: Age and treatment were significant predictors of SaO2/FiO2_10_ (F = 10.75, *p* < 0.001; R^2^ = 0.24, treatment: β = 0.33, t = 3.83, *p* < 0.003; age: β = −0.40, t = −3.09, *p* = 0.003).

All these regression values showed that treatment is the primary variable in determining differences in the outcomes.

### 3.6. Safety

The rate of severe complications, including bacteremia, nosocomial pneumonia, gastrointestinal bleeding, and severe tachyarrhythmias, was not higher among patients who received HDCPT vs. those who received SCT. Following the use of high-dose corticosteroids, most of the patients required insulin due to their known or hidden diabetes. However, the insulin requirement level was controlled by the doctors and returned to normal at discharge, and there were no adverse events according to uncontrolled diabetes in the patients. In addition, the site investigators determined that patients who did not have significant side effects other than hyperglycemia during therapy, as well as other events during the research, were not related to high-dose steroid treatment.

## 4. Discussion

The present study aimed to compare the association of two different regimens of steroid therapy combined with antiviral therapy in patients with severe COVID-19 pneumonia. We prospectively investigated in patients with severe COVID-19 pneumonia treated with remdesivir whether an early intensive course of glucocorticoid reduces mortality and improves clinical outcomes, particularly inducing a faster improvement in the SaO2/FiO2 index.

We hypothesized early combined therapy including remdesivir, an antiviral agent that inhibits viral RNA-dependent RNA polymerase, and a pulse dose of methylprednisolone (MP), designed to counteract the increase in cytokine storm virus-induced cytokine storm, can offer a significant benefit in recovery and mortality in hospitalized patients with severe COVID-19. Additionally, we hypothesized that the administration time and dose of steroids are critical factors in the efficacy of corticosteroid treatment.

Our study found that early combination treatment with high-dose pulses of methylprednisolone and remdesivir significantly decreased mortality and improved lung involvement in patients with severe COVID-19 pneumonia. The reduction in death rate was caused by lower demand for mechanical breathing and ICU hospitalization.

Remdesivir and prednisone are currently the most extensively used evidence-based therapy in patients with COVID-19 pneumonia [3,4,5,6,8]. Previous studies showed a positive impact of remdesivir treatment on the clinical outcomes of hospitalized patients with COVID-19 [6,7]. Unfortunately, antiviral therapy success in patients with severe COVID-19 pneumonia remains limited. Severe COVID-19 is characterized by a hyper-inflammatory response with overproduction of early response pro-inflammatory cytokines that can lead to multi-organ failure and death. Thus, in patients with severe COVID-19 pneumonia, early and effective anti-inflammatory treatment is essential to avoid the progression of COVID-19 to a critical stage.

Corticosteroids exert anti-inflammatory activities. Previous studies showed the benefits of corticosteroid therapy in patients with COVID-19 pneumonia, and the efficacy of corticosteroids seems to be associated with the most severe stages of COVID-19 disease [8,9,10].

However, even if the effectiveness of glucocorticoid therapy in reducing mortality in COVID-19, especially in severe patients is demonstrated, the optimal time and dose for administering steroids to receive the maximum benefits are undetermined. Corticosteroid pulses have been suggested to show higher clinical benefits than low-dose corticosteroids because they act on different pathways that perform genomic and non-genomic functions [18,19] with the expectation of strong suppression of the cytokine storm that prevents the organization of pneumonia and lung fibrosis. In a published randomized controlled trial of pulsed methylprednisolone therapy, there was a survival benefit in patients with COVID-19 pneumonia [11]. Other retrospective non-randomized studies suggest that corticosteroids pulse therapy was associated with a survival benefit and a reduction in the need for intubation in hospitalized patients with COVID-19 [12,13,14,20,21] and improved late outcomes three months after discharge [22].

Furthermore, a recent meta-analysis showed that pulse doses of corticosteroids lower mortality and the need for MV in patients with COVID-19 without significantly increasing severe adverse events [23]. Criteria to identify the clinical and radiological characteristics of the patients most likely to benefit from pulsed methylprednisolone therapy have also been suggested [24].

Current guidelines recommend using remdesivir and a low dose of systemic corticosteroids in patients with COVID-19 pneumonia and respiratory failure [3,5].

In patients with severe COVID-19, combination therapy with an antiviral and an anti-inflammatory agent may dampen the potentially harmful inflammatory response through its etiologic and immunomodulating effects. However, there is limited data and clinical studies assessing the potential benefits on clinical outcome of the combination of remdesivir plus dexamethasone compared with remdesivir alone [25,26,27], showing better clinical outcome in patients treated with combined therapy.

Moreover, the optimal dose and timing of steroids in patients treated with remdesivir are unclear.

In most previous studies, corticosteroid doses were low (dexamethasone 6 mg per day or equivalent) or intermediate (dexamethasone up to 20 mg per day or equivalent) [8,9,10]. Moreover, several studies assessing corticosteroid pulse versus low-dose corticosteroids for patients with COVID-19 have been reported, many with favourable results on clinical outcomes [11,12,13,14], particularly in patients with severe or critical COVID-19. However, robust evidence to address whether pulse corticosteroids are superior to low-dose corticosteroids for patients with severe COVID-19 is lacking.

This is the first study investigating the effectiveness of combined therapy with remdesivir plus methylprednisolone pulse. The present study enrolled a severely ill population of patients with COVID-19 in the early pulmonary phase. The mortality rate was significantly lower among patients treated with remdesivir plus HDCPT than those treated with remdesivir plus standard steroid treatment.

Furthermore, patients treated with remdesivir plus HDCPT presented higher SaO2/FiO2 values at five and ten days and lower admission to intensive cure and mortality than those who received remdesivir plus SCT. The strength of our data was indicated by regression analyses that clearly showed the significant impact of treatment on mortality at 30 days, admission to the ICU, and SaO2/FiO2 at 5 and 10 days. Although age was a significant variable in determining SAO25 and SAO210, its weight on these variables was lower than the treatment typology. Moreover, only the treatment typology significantly affected NIV, ICU admission, and exit. Therefore, our data strongly suggest that combination treatment with MP pulse and remdesivir can improve clinical outcomes in hospitalized patients with severe COVID-19 pneumonia.

The complication induced by the treatment with methylprednisolone is a major concern, particularly secondary infections. However, no severe complications caused by methylprednisolone treatment were observed in the present study, including nosocomial infections.

The present work has some limitations. First, the monocentric nature, the limited sample size of the study, and the inherent limitations of an observational study can affect the generalizability of our results. Second, different therapies from the corticosteroid regimen were administered according to the treating physician’s decision and without prespecified criteria. Third, the lack of late follow-up to identify late adverse events (e.g., hip osteonecrosis).

Despite the current study’s limitations, we think that our results raise the possibility that early combined therapy of remdesivir plus pulsed methylprednisolone can be an effective treatment option in patients with severe COVID-19 pneumonia. Compared with novel therapeutic agents such as pricey monoclonal antibodies and targeted molecular pharmaceuticals with limited and restricted access, this combined treatment may be more readily available and affordable worldwide.

## 5. Conclusions

In conclusion, in this study early administration of HDCPT plus remdesivir was associated with lower mortality at 30 days, reduced admission to the ICU and the need for IMV compared with standard treatment. However, more randomized studies or extensive data analyses are required to confirm if combination treatment with pulse methylprednisolone and remdesivir can help improve clinical outcomes in hospitalized patients with severe COVID-19 pneumonia and to determine the type of COVID-19 patients who are optimally suited for higher doses of corticosteroids.

## Figures and Tables

**Table 1:** Baseline demographics and clinical characteristics of study patients.

	Treatment	M	SD	t
Age	R + SCT	64.38	14.27	0.24 (*p* = 0.81)
	R + HDCPT	63.75	12.41	
Comorbidity (CSI)	R + SCT	3.11	2.09	−0.28 (*p* = 0.77)
	R + HDCPT	3.22	1.86	
SaAO2/FiO2_0_	R + SCT	207.30	44.17	1.14 (*p* = 0.25)
	R + HDCPT	197.35	43.31	

**Table 2:** Need for admission to the ICU with invasive ventilation and NIV in the Remdesivir + HDMPT and Remdesivir + SCT groups.

Outcome	Treatment	*n*/Total *n* (%)	*p*-Value
ICU admission and invasive ventilation	R + SCT R + HDCPT	14/47 (29.8%) 4/55 (7.3%)	0.04
NIV	R + SCT R + HDCPT	30/47 (63.8%) 17/55 (30.9%)	<0.001

**Table 3:** Impact of treatment typology, age and number of comorbidities on SAO_2_ at days five and ten, invasive care unit admission, and death.

SAO2_5_	F = 5.518, *p* = 0.002; R^2^ = 0.14		
	Standard *β*	t	*p*
Treatment	0.28	3.04	0.003
Age	−0.34	−2.46	0.01
Number of comorbidities	0.22	1.55	0.12
**SAO2_10_**	**F = 10.75, *p* < 0.001; R^2^ = 0.24**		
	Standard *β*	t	*p*
Treatment	0.33	3.83	< 0.001
Age	−0.40	−3.09	0.003
Number of comorbidities	0.07	0.58	0.56
**NIV**	**F = 5.66, *p* < 0.001; R^2^ = 0.48**		
	Standard β	t	*p*
Treatment	−0.32	−3.45	< 0.001
Age	0.22	1.58	0.11
Number of comorbidities	−0.03	−0.25	0.80
**ICU Admission**	**F = 4.25, *p* < 0.05, R^2^ = 0.11**		
	Standard *β*	t	*p*
Treatment	−0.29	−3.06	0.003
Age	0.14	0.99	0.32
Number of comorbidities	0.03	0.23	0.82
**EXITUS**	**F = 7.55, *p* < 0.001; R^2^ = 0.18**		
	Standard *β*	t	*p*
Treatment	−0.31	−3.41	0.001
Age	0.23	1.73	0.08
Number of comorbidities	0.07	0.53	0.59

## Data Availability

The data presented in this study are available on request from the corresponding author. The data are not publicly available due to the protection of patient privacy.
